# Construction of recombinant *Lactococcus* expressing thymosin and interferon fusion protein and its application as an immune adjuvant

**DOI:** 10.1186/s12934-024-02308-1

**Published:** 2024-02-06

**Authors:** Zengqi Liu, Suhua Zhang, Hongjiao Hu, He Wang, Yu Qiu, Mingqi Dong, Muping Wang, Ziyang Cui, Hongyu Cui, Yunfeng Wang, Gaoming He

**Affiliations:** 1 College of Animal Science and Technology, Shihezi University, Shihezi, 832003 Xinjiang China; 2 Harbin Guosheng Biotechnology Co., Ltd, Harbin, 150028 China; 3 Clinical Medical College, Hebei North University, Zhangjiakou, 075000 China; 4 State Key Laboratory of Veterinary Biotechnology, Harbin Veterinary Research Institute, Chinese Academy of Agricultural Sciences, Harbin, 150069 China

**Keywords:** Thymosin, Interferon, Recombinant *Lactococcus*, Immune enhancer, Immune adjuvant

## Abstract

**Background:**

In recent years, biosafety and green food safety standards have increased the demand for immune enhancers and adjuvants. In the present study, recombinant food-grade *Lactococcus lactis* (r-*L. lactis*-Tα1-IFN) expressing thymosin Tα1 and chicken interferon fusion protein was constructed.

**Results:**

The in vitro interactions with macrophages revealed a mixture of recombinant r-*L. lactis*-Tα1-IFN could significantly activate both macrophage J774-Dual™ NF-κB and interferon regulator (IRF) signaling pathways. In vitro interactions with chicken peripheral blood mononuclear cells (PBMCs) demonstrated that a mixture of recombinant r-*L. lactis*-Tα1-IFN significantly enhanced the expression levels of interferon (IFN)-γ, interleukin (IL)-10, CD80, and CD86 proteins in chicken PBMCs. Animal experiments displayed that injecting a lysis mixture of recombinant r-*L. lactis*-Tα1-IFN could significantly activate the proliferation of T cells and antigen-presenting cells in chicken PBMCs. Moreover, 16S analysis of intestinal microbiota demonstrated that injection of the lysis mixture of recombinant r-*L. lactis*-Tα1-IFN could significantly improve the structure and composition of chicken intestinal microbiota, with a significant increase in probiotic genera, such as *Lactobacillus spp*. Results of animal experiments using the lysis mixture of recombinant r-*L. lactis*-Tα1-IFN as an immune adjuvant for inactivated chicken Newcastle disease vaccine showed that the serum antibody titers of the experimental group were significantly higher than those of the vaccine control group, and the expression levels of cytokines IFN-γ and IL-2 were significantly higher than those of the vaccine control group.

**Conclusion:**

These results indicate that food-safe recombinant r-*L. lactis*-Tα1-IFN has potential as a vaccine immune booster and immune adjuvant. This study lays the foundation for the development of natural green novel animal immune booster or immune adjuvant.

## Introduction

Immune enhancers are substances that are unrelated to antigens and can augment the body´s immune response when used alone or simultaneously with antigens, whereas immune adjuvants are substances that can be used in combination with vaccines to improve the immune response to vaccine antigens [1]. Studies have shown that several substances exist with immune-enhancing effects, such as oil-water emulsions, microorganisms and their metabolites, nucleic acids and their analogs, cytokines, propolis, immunostimulatory complexes, liposomes, and artificial chemically synthesized molecules. However, these substances face many challenges, such as the induction of disorders, allergic reactions, hypersensitization, and cytotoxicity [2, 3]. In recent years, biosafety and green food safety have placed high demands on future biologics, especially on the biosafety of preventive biological agents. Therefore, research has focused on natural peptides and proteins derived from animals as immune adjuvants or enhancers.

Natural immune peptides, which are mostly peptides with natural immunomodulatory activity obtained from animals or food proteins [4], can regulate specific and non-specific immune functions by affecting the growth and development of immune organs (tissues), proliferation and transformation of lymphocytes, and/or release of immune molecules (cytokines and antibodies). Natural immune peptides play important roles in maintaining immune function and health [5]. As they are natural proteins derived from animals, no biosafety or food safety concerns exist.

Thymic peptides are a class of peptides secreted by the epithelial cells of the thymus and are involved in immune development and regulation. Among them, thymosin Tα1 is a short peptide consisting of 28 amino acids and is highly conserved among different animal species [6, 7]. Thymosin Tα1 has important immunomodulatory activities [8]: the peptide can promote T cell proliferation, and secretion of various cytokines by natural killer (NK) cells, such as interleukin-2 (IL-2) and interferon-α (IFN-α). Moreover, thymosin Tα1 is an immune enhancer against CD_3_^+^-induced apoptosis and enhances NK cell function, inhibits tumor growth, and performs a series of other immunomodulatory functions [9]. At present, the thymus peptides used in veterinary clinical practice are mostly extracted from bovine thymus tissue and are mostly a mixture of small peptides, which have various concerns, such as large content of impurities, low Tα1 content (about 0.1%), poor treatment effect, requirement for large dosage, serious side reactions, and other problems; furthermore, the extraction of peptides from the thymus is challenging due to low yield [5]. Purified synthetic Tα1 products are also available; although they are expensive. Therefore, exploring the use of genetic engineering methods to obtain highly active Tα1 at low cost has become the first choice.

IFN, discovered in 1957 as a substance that interferes with viral reproduction, is a natural protein produced by the immune system of most vertebrates when resisting viruses, bacteria, parasites, tumor cells, and other foreign substances. IFN-γ is mainly produced by activated T cells and natural killer cells and is involved in immune regulation and inflammatory responses, with critical immunomodulatory and antiviral effects. In recent years, some studies have confirmed that the combined application of interferon and thymosin synergistically promotes T cell growth, proliferation, differentiation, and activation of NK cell activity, which can enhance the body´s immune capacity [10, 11]. The combination of IFNs and thymosin as natural immune enhancers or immune adjuvants has brought new hope for the treatment of viral and neoplastic diseases [12].

However, natural peptides obtained from animal tissues face many difficulties, such as difficulties in extracting sufficient amounts and purification. Moreover, the use of genetic engineering to express natural proteins is challenging. Many scientists have used *Escherichia coli* and yeast to express natural proteins; however, their applications are limited by the presence of substances such as endotoxins. Food-grade lactic acid bacteria genetically engineered to express natural proteins have become the first choice because they do not contain endotoxins. Therefore, they can completely replace *E. coli* as live vectors for delivering proteins of interest in food or in vivo. They are the most commonly used gram-positive host bacteria and gene expression systems. Lactic acid bacteria have been widely and successfully used in food fermentation for thousands of years [13]. Most lactic acid bacteria species are beneficial to animals, plants, and humans; as probiotics, they are not only endotoxin-free, but their cell wall and nucleic acid and other components possess immune adjuvant activity [14]. Among the many benefits of probiotics, their ability to modulate the immune system occupies an important position [15], and several studies have provided clear evidence of the modulatory properties of certain probiotics on the immune system [16]. Furthermore, some probiotics can enhance the non-specific immune functions of the organism, including the phagocytic activity of innate immune cells and the cytotoxicity of NK cells [16], and also enhance the specific immune response function of the organism, promoting the stimulation of B cells to secrete specific IgA and IgG and activating helper T lymphocytes and macrophages [17, 18]. Amidst the current critical scenario of food safety and biosafety, utilizing food-grade lactic acid bacteria, with their inherent safety features, becomes the preferred choice for developing immune adjuvants and enhancers. This choice aligns with their safety and probiotic functions.

In the present study, recombinant *Lactococcus lactis* (r-*L. lactis*-Tα1-IFN) expressing chicken thymosin Tα1 and chicken IFN-γ fusion protein was constructed using food-grade *Lactococcus lactis* NZ3900 and an antibiotic-free labeled expression vector. In vitro and in vivo experiments demonstrated that the lysis mixture of r-*L. lactis*-Tα1-IFN had good immune booster and immune adjuvant activities. The findings of this study provide a strong foundation for the research and application of green food-safe grade vaccine adjuvants or immune enhancers.

## Materials and methods

### Bacterial strains, cells, and reagents

The host strain *Lactococcus lactis* NZ3900 and the expression vector pNZ8149 were purchased from MoBiTec (Gottingen, Germany). Macrophage J774-Dual™ (InvivoGen, Hong Kong) cells were derived from the mouse J774.1 macrophage-like cell line. The cells were engineered by stably integrating two reporter genes (alkaline phosphatase (AKP) and Lucia luciferase, five reporter genes were inserted into the cellular genome according to the instructions for Macrophage J774-Dual™ respectively). J774-Dual™ cells can be used to detect activation of the NF-kB pathway by assessing the expression of the AKP reporter and monitoring luciferase activity to assess the activation of the interferon regulatory factor (IRF) pathway. Dulbecco’s modified Eagle’s medium (DMEM) was obtained from Thermo Fisher Scientific (USA). J774-Dual™ cells were maintained in DMEM (supplemented with 10% heat-inactivated Fetal Bovine Serum (FBS), 1% penicillin-streptomycin, 100 µg/mL Normocin, 100 µg/mL Zeocin (InvivoGen, France), and 5 µg/mL Blasticidin (InvivoGen, France)). Lipopolysaccharide, 2’3’-cGAMP, and Pam3CSK4 were obtained from InvivoGen. Chicken peripheral blood lymphocyte separation solution kit was purchased from TBD (China). Additionally, FBS was purchased from Gibco (USA). Roswell Park Memorial Institute (RPMI) 1640 medium was obtained from Sigma (USA); Phorbol-12-myristate-13-acetate (PMA), Ionomycin, and ConA were purchased from InvivoGen (Toulouse, France). DNA gel extraction and purification kits were purchased from Axygen. The one-step cloning kit was purchased from Vazyme Biotechnology (Nanjing, China). PrimerStar Max, SYBR Green Real-Time polymerase chain reaction (PCR) Master Mix, and Premix Ex Taq (Probe qPCR) kits were purchased from Toyobo Biotechnology Co., Ltd. Ex Taq enzyme and RNAiso Plus were purchased from TaKaRa. Anti-HA-tagged mouse monoclonal antibodies were purchased from Beijing Bolong Company. The Newcastle disease oil emulsion-inactivated vaccine (NDV Lasota strain) was purchased from Harbin Weike Biology Co., Ltd.

### **Construction of recombinant plasmids and recombinant r-*L. lactis-*Tα1-IFN**

The fusion protein was designed based on the reference sequences of thymosin Tα1 and chicken interferon from GenBank, and the DNA sequence was synthesized via gene codon optimization by Genescript, Inc (Nanjing, China) based on the genome of the host strain NZ3900 (fusion protein with HA tag at the carboxyl end). The fusion gene was amplified via PCR using forward primer 5’-ACCATGGGTACTGCAGGCATG-3’ and reverse primer 5’-AGCTCTCTAGAACTAGTGGTACC-3,’ according to One Step Cloning Kit (Vazyme Biotechnology). Homologous recombination was performed and cloned into plasmid pNZ8149. The cloned region was sequenced after each construction stage, and the final recombinant plasmid was electrotransformed into the competent receptor host *Lactococcus lactis* NZ3900. Bacteria were cultured in plates coated with Elliker medium for the selection of Lac + transformants. Ingredients included: 20 g/L tryptone, 5 g/L yeast extract, 4 g/L sodium chloride, 1.5 g/L sodium acetate (anhydrous), 0.5 g/L L-(+) ascorbic acid) agar containing 0.5% lactose to select lactose-resistant clones. The cultures were incubated at 30 °C for 24 h according to a standard protocol [19]. Subsequently, the r-Tα1-IFN fragment was detected via PCR to identify a positive recombinant strain.

### Western blot analysis

The constructed recombinant *Lactococcus* was inoculated into the L-Elliker liquid medium at a ratio of 1:100. When the Optical Density (OD) value was approximately 0.35, the inducer nisin was added to a final concentration of 20 ng/mL, and bacteria were incubated for 6 h at 30 °C, washed twice with phosphate buffer saline (PBS), and then concentrated 10-fold. After ultrasonic crushing using an ultrasonic crusher, the supernatant was collected and centrifuged at 3,000×g for 10 min. *Lactococcus lactis* NZ3900 containing the control pNZ8149 plasmid, as previously described, served as the wild-type wt-*L. lactis* control. The supernatants were analyzed using western blotting. The specific steps include: Following separation in a 12% SDS-PAGE gel, proteins were electrically transferred to a nitrocellulose membrane, which was blocked at room temperature (25 ℃) with 5% skimmed milk for 2 h. Membranes were washed thrice with PBS-Tween-20 (PBST) and incubated with HA murine monoclonal antibody (1:2000) at room temperature (25 ℃) for 1.5 h. Following four washes with PBST, membranes were incubated with sheep anti-mouse infrared labeled secondary antibody at room temperature for 50 min, washed thrice with PBST, and imaged using an Odyssey Infrared Scanner.

### Quantification of recombinant Tα1-IFN protein and preparation of recombinant protein mixture

After induction of the expression of the recombinant protein in lactic acid bacteria according to the aforementioned method, the bacteria were washed three times with sterile PBS solution, and the OD600 value of the bacterial solution was adjusted to 2 using PBS (pH 8.5) (the concentration of lactic acid bacteria was approximately 2 × 10^8^ CFU/mL). The bacteria were then ultrasonically disrupted (the ultrasonication time was adjusted to 18 min) and centrifuged at 12,000×g for 10 min. The supernatant was collected and filtered with a 0.45-µm filter. The recombinant proteins were quantified using the bicinchoninic acid assay (BCA) method. The supernatant of the recombinant protein mixture was stored at – 80 °C.

### Detecting the immune activation properties of the lysis mixture of recombinant Tα1-IFN protein on macrophages in vitro

The macrophage cell line J774-Dual™ (Invivogen) was cultured at 37 °C in a 5% carbon dioxide (CO_2_) incubator in DMEM containing 100 mg/mL Normocin, 100 U/mL penicillin, 100 µg/mL streptomycin, 5 µg/mL Blasticidin, and 100 µg/L Zeocin (Thermo Fisher, USA). Cell scrapers were used to detach and count the cells. The cells were centrifuged at 300×g for 5 min. The supernatant was removed, and J774-Dual™ cells were resuspended in fresh, pre-warmed growth medium at 2.8 × 10^5^ cells/mL.

To detect the activation of the NF-κB signaling pathway in J774-Dual™ cells, 100 µL of J774-Dual™ at a density of 2.8 × 10^5^ cells/mL was seeded in 96-well cell culture plates, and 20 µL (27 µg/mL) of the prepared recombinant Tα1-IFN recombinant protein mixture was added to each well. Pam3CSK4 was used as a positive activation control, and 20 µL PBS was used as a negative control. The cells were incubated at 37 °C in a 5% CO_2_ incubator for 24 h, and the culture supernatant was collected and stored at -80 °C for later use. Then, 170 µL of the QUANTI-Blue™ solution (InvivoGen, France) was added to each well of a 96-well plate, followed by 30 µL of the J774-Dual™ cell supernatant, and incubated at 37 °C in a 5% CO_2_ incubator for 4–8 h. The NF-κB-induced AK expression level was measured at 620–655 nm using a microplate spectrophotometer (ELx808, Bio Tek Instruments, Inc. USA).

Detection of interferon regulatory factor (IRF) signaling pathway activation in J774-Dual cells. J774-Dual™ cells (100 µL at a density of 2.8 × 10^5^ cells/mL) were seeded into a 96-well cell culture plate, and 20 µL (27 µg/mL) of prepared Tα1-IFN recombinant protein mixture was added to each test well. 2’3’-GAMP was used as a positive control, and 20 µL of PBS as the negative (non-stimulating) control. A 20 µL sample of lysed *Lactococcus lactis* NZ3900 (wt-*L. lactis*, same number of bacteria as 20 µL of prepared recombinant Tα1-IFN protein mixture) was used as wt-*L. lactis* control. After stimulating the cells at 37 °C in a 5% CO_2_ incubator for 24 h, the culture supernatant was collected. A sample of 20 µL was added per well to the white (opaque) 96-well plates, and luciferase expression was determined using a microplate chemiluminescence detector (Centro XS^3^ LB 960, Berthold Technologies, Bad Wildbab, Germany) with the following photometer parameters: 50 µL QUANTI-Luc™ injection, endpoint measurement, start time 4 s, end reading 0.1 s, followed by measurement.

### Detection of activated PBMCs by recombinant Tα1-IFN protein lysate in vitro

According to the instructions of the chicken peripheral blood lymphocyte isolation solution kit (TBD), the PBMCs were isolated from the anticoagulant-treated blood samples of chickens and resuspended in RPMI 1640 culture medium (Sigma) containing 150 µg/mL gentamicin, 100 µg/ml Normocin, 2 mM-glutamine, and 10% heat-inactivated FBS (Gibco, USA) at 37 °C. Moreover, the cell concentration was adjusted to 1 × 10^6^ cells/mL for subsequent use. PBMCs (1 mL at a density of 1 × 10^6^ cells/mL) were seeded into a 24-well cell culture plate, and 20 µL (27 µg/mL) of the prepared recombinant Tα1-IFN protein mixture was added. A 50-µL sample of supernatant of lysed *Lactococcus lactis* NZ3900 (wt-*L. lactis*, same number of bacteria as 20 µL of prepared recombinant Tα1-IFN protein mixture) was used as wt-*L. lactis* control. After stimulation for 24 h at 37 °C in a 5% CO_2_ incubator, cells were collected, centrifuged, and stored at -80 °C for backup. RNA was extracted as follows: cells were harvested, centrifuged, and extracted using RNAiso Plus (TAKARA) according to the manufacturer’s instructions. Reverse transcription of RNA to complementary DNA was performed using reverse transcriptase (BioRt). Additionally, RT-qPCR was performed using SYBR qPCR Mix (Vazyme) according to the manufacturer’s instructions (see Table 1 for primers). The transcript levels of IFN-γ, IL-10, CD80, and CD86 were quantified, and the results were analyzed by the ΔΔCT method.

**Table 1:** Primer sequences

Primer	Primer sequences (5’- 3’)
IFN-γ-F	CTCCCGATGAACGACTTGAG
IFN-γ-R	CTGAGACTGGCTCCTTTTCC
IL-10-F	CTGTCACCGCTTCTTCACCT
IL-10-R	ACTCCCCCATGGCTTTGTA
CD80-F	CAGCAAGCCGAACATAGAAAGA
CD80-R	AGCAAACTGGTGGACCTGAGA
CD86-F	TACCTTGGCCAGGAAAAACA
CD86-R	ATACTGCCCCTCATCCACAA
β-actin -F	TCCACCGCAAATGCTTCTAAAC
β-actin -R	CTGCTGACACCTTCACCATTCC

### Detection of proliferative activity of T lymphocytes in PBMCs

The isolated chicken peripheral blood lymphocytes were washed twice with fresh RPMI 1640 medium and resuspended at a density of 1 × 10^6^ cells/mL in RPMI 1640 medium containing 10% FBS, 100 U/mL penicillin, 100 µg/mL streptomycin, 100 µg/mL Normocin, and 100 µg/mL gentamicin. Then, 100 µL of cells were added to 96-well plates with a mixture of Ionomycin (Ionomycin, 3 µg/mL, Sigma) and phorbol 12-myristate 13-acetate (PMA, 100 ng/mL) as stimuli. The plates were incubated at 37 °C in a 5% CO_2_ incubator for 48 h, and 10 µL of Cell Counting Kit-8 (CCK-8; Dojindo, Japan) was added per well for 4 h. Absorbance was measured at 450 nm using a microplate spectrophotometer (ELx808, Bio Tek Instruments, Inc. USA).

### Animal experimental design and selection of experimental chickens

Specific pathogen-free (SPF) chickens (White Laihang Chickens) were purchased from the Harbin Veterinary Research Institute, Chinese Academy of Agricultural Sciences (Harbin, China) and housed in a negative-pressure filtered air isolator at Harbin Guosheng Biotechnology Co. (Harbin, China). All animal experiments were conducted in accordance with the specifications for laboratory animals provided by the Ministry of Science and Technology of the People’s Republic of China (Beijing, China). All SPF chickens were reared and handled in accordance with the principles of human care and laboratory animal use.

Experimental procedure for the administration of r-*L. lactis*-Tα1-IFN as an immune booster in chickens: 21-d-old SPF chickens were randomized into three groups: a blank control group, wt-*L. lactis* control group, and r-*L. lactis*-Tα1-IFN immune booster group (Fig. 3a). Ten animals in each group were immunized using injection at 21 and 28 days of age. The r-*L. lactis*-Tα1-IFN immune booster group was injected intramuscularly with 200 µL recombinant protein mixture (27 µg/mL) per animal. Additionally, a 200 µL sample of lysed *Lactococcus lactis* NZ3900 (wt-*L. lactis*, the same number of bacteria as 200 µL of prepared recombinant Tα1-IFN protein mixture) was used as wt-*L. lactis* control. The blank control group was injected intramuscularly with 200 µL PBS per animal. Blood, serum, and anticoagulated blood were collected during the 1st, 2nd, and 3rd weeks after immunization (the 2nd, 3rd, and 4th weeks after the first vaccination). Peripheral PBMCs were prepared for the detection of proliferative activity, and serum cytokine detection was performed as described below (Fig. 3a). The chickens were euthanized at week 7 post-immunization, and samples of cecal intestinal contents were collected for 16S ribosomal RNA (rRNA) analysis, as described below.

Preparation of water-in-oil (W/O) immunoadjuvant: The aqueous phase consisting of r-*L. lactis*-Tα1-IFN mixture with 0.25% surfactant Tween-80 was shaken well in a warm bath at 37 °C for 30 min; the oil phase was prepared by adding 2% aluminum stearate to white oil (10# white oil) with 5% Span-80 and was autoclaved. The emulsion consisted of two parts of the oil phase (oil emulsion) and one part of the water phase, which were mixed and emulsified to form a W/O emulsion for preparing r-*L. lactis*-Tα1-IFN immunoadjuvant oil emulsion.

Preparation of r-*L. lactis*-Tα1-IFN immunoadjuvant for Newcastle disease oil emulsion inactivated vaccine: One part of r-*L. lactis*-Tα1-IFN immunoadjuvant oil emulsion was added to one part of the chicken Newcastle disease oil emulsion-inactivated vaccine and re-emulsified by an emulsifier to obtain r-*L. lactis*-Tα1-IFN immunoadjuvant Newcastle disease oil emulsion inactivated vaccine.

Experimental procedure for the injection of r-*L. lactis*-Tα1-IFN as an immune adjuvant for inactivated Newcastle disease vaccine: SPF chickens at 14 days old were randomly divided into four groups of 10 chickens each: blank control group, vaccine control group, wt-*L. lactis*-adjuvant control group and r-*L. lactis*-Tα1-IFN oil emulsion-inactivated vaccine test group. The r-*L. lactis*-Tα-IFN immune adjuvant Newcastle disease oil emulsion-inactivated vaccine test group and wt-*L. lactis* adjuvant control group were injected with 0.6 mL oil emulsion per animal; the Newcastle disease oil emulsion-inactivated vaccine control group was injected with 0.3 mL Newcastle disease oil emulsion-inactivated vaccine per animal (to ensure that the antigen dose in the immune adjuvant group was the same as that in the vaccine control group); the blank control group was injected with 0.3 mL of PBS. Blood was collected on days 3, 7, and 14 d after immunization for serum preparation, antibody, and cytokine testing. Furthermore, PBMCs were collected for the detection of proliferative activity on day 14 after immunization.

### Analysis of intestinal microbiota 16S diversity

Chinese white Laihang SPF chickens in the experimental group were euthanized separately from those in the control group. To minimize animal suffering, euthanasia was performed using deep anesthesia (Zoletil 50), followed by bloodletting. In addition to collecting serum and other organs, samples of cecum intestinal contents were also collected, placed in 15 mL centrifuge tubes, and quickly stored in a − 80 °C freezer for cryopreservation and transported in a dry ice storage box to a sequencing company (Shanghai Meiji Biotechnology Co., Ltd., China) for sample DNA extraction. The primers used for amplification of the V3–V4 region of the 16S rRNA gene were forward primer 343 F and reverse primer 798R. All samples were analyzed using the Megasound Cloud Analysis Platform (Shanghai Megasound Biotechnology Co., Ltd., China).

### Hemagglutination inhibition (HI) test

The serum to be tested was removed from − 20 ℃ and set aside. First, the Hemagglutination (HA) titer of NDV virus antigen was detected: the Lasota strain NDV virus antigen solution was diluted sequentially in a 96-well hemagglutination plate, and 50 µL of the prepared 0.5% chicken red blood cell suspension was added to each well, placed in a 37 °C incubator for 30 min, and the HA titer was read. Based on the HA titer of the antigen, a viral liquid was prepared from eight and four HA units (HAU). A 50-µL aliquot of eight HAU of the virus was added to the first column of the 96-well HA plate, and 50 µL of four HAU virus solutions were added to each of the remaining wells. The 50 µL of the tested serum was added to the first well, serum was diluted one by one, then, 50 µL of 0.5% chicken red blood cell suspension was added to all wells, mixed gently, and placed in a 37 °C incubator for 30 min before reading the results. Antibody titers were expressed as 1:2^n^ (n is the highest dilution of the wells in which the agglutination of erythrocytes inhibited by the serum was 100%), and serum titers were expressed as Log2 of the reciprocal of the highest antibody dilution inhibiting four HAU of the virus.

### Detection of cytokines

Detection of serum cytokines: The collected serum was diluted at a ratio of 1:2, and the levels of IFN-γ, IL-2, IL-4, and IL-10 were determined using an enzyme-linked immunosorbent assay (ELISA) kit (Solarbio, Beijing, China). OD values were measured at 450 nm. Cytokine concentrations were calculated according to the standard curve and the manufacturer’s instructions.

### Data statistical analysis

Ordinary one-way analysis of variance was performed using the Graph PadPrism 10.0 software (GraphPad, La Jolla, CA, USA) to detect differences between groups. Statistical significance was set at *P < 0.05, **P < 0.01, ***P < 0.001, and ****P < 0.0001.

## Results

### **Construction of recombinant *Lactococcus *and induced expression of the Tα1-IFN protein**

**Fig. 1:**
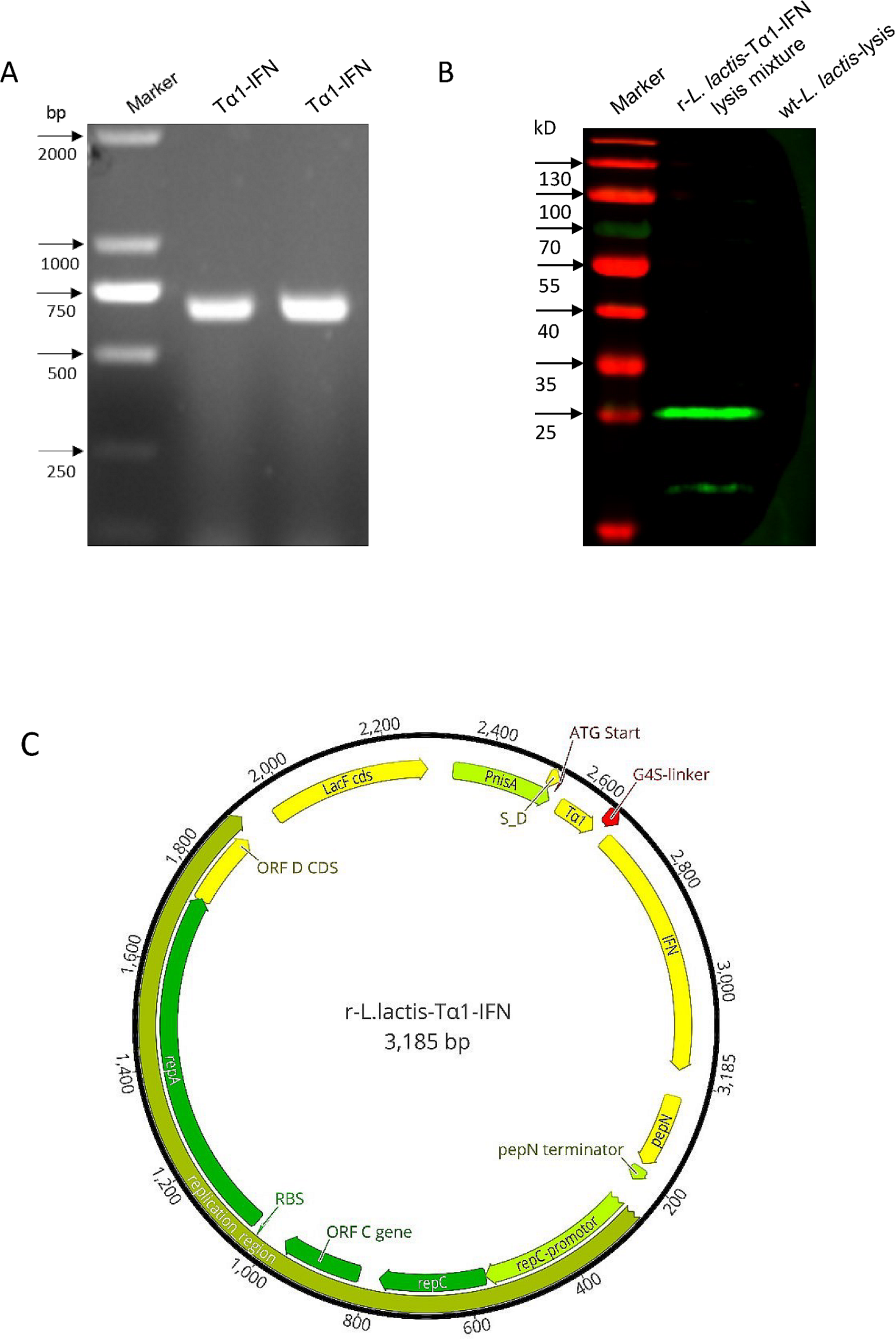
Construction of a recombinant plasmid pNZ8149-Tα1-IFN expressing Tα1-interferon fusion protein. **(a)** The recombinant plasmid pNZ8149-Tα1-IFN was identified via PCR to amplifies Tα1-IFN fusion gene (674 bp); **(b)** Identification of recombinant protein Tα1-IFN via western blotting for recombinant lactic acid bacteria r-*L. lactis*-Tα1-IFN; **(c)** Plasmid map of recombinant plasmid pNZ8149-Tα1-IFN.

The recombinant plasmid pNZ8149-Tα1-IFN was identified via PCR to amplify a 674-bp gene fragment (Fig. 1a, lanes 2 and 3), which was sequenced completely. The recombinant *Lactococcus lactis* (r-*L. lactis*-Tα1-IFN) was obtained by electrotransferred pNZ8149-Tα1-IFN plasmid into *L. lactis* NZ3900, and recombinant *Lactococcus lactis* (r-*L. lactis*-Tα1-IFN) was induced to be expressed. The soluble target protein Tα1-IFN was expressed in the cytoplasm. However, the protein was not secreted into the culture medium, and was not anchored to the cell wall (data not displayed). Western blotting yielded a recombinant Tα1- IFN that matched the size of the intended target recombinant protein (approximately 25 KD) (Fig. 1b, lane 2), and the concentration of r-Tα1-IFN protein was determined to be 27 µg/mL using a BCA assay.

### **The lysis mixture of r-*L. lactis*-Tα1-IFN activated IRF and NF-κB signaling pathways in macrophages in vitro**

**Fig. 2:**
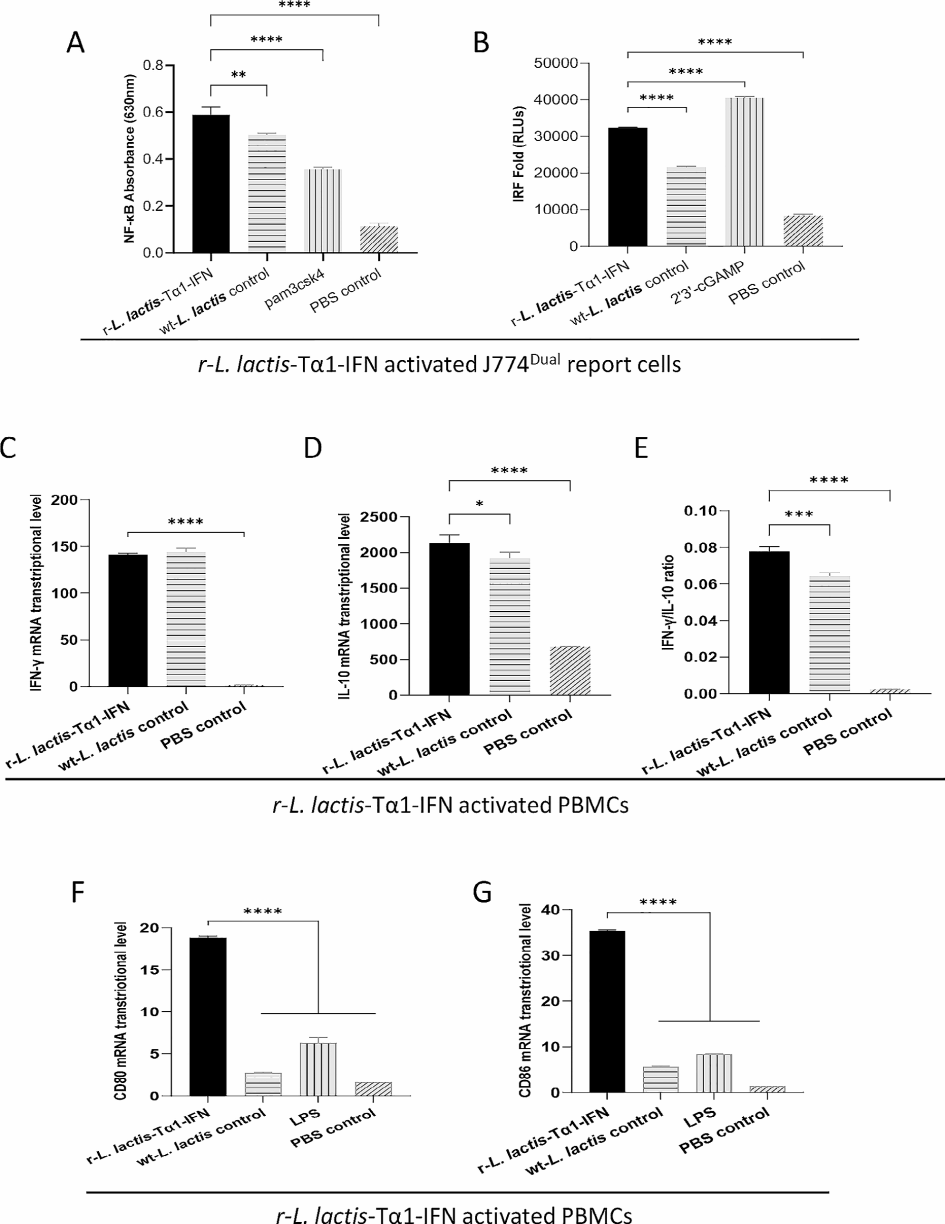
Recombinant r-*L. lactis*- Tα1-IFN activates macrophages J774-Dual™ cells and peripheral blood mononuclear cells (PBMCs). **(a)** Detection of activation of NF-κB signaling pathway in J774-Dual™ reporter cells; **(b)** Detection of activation of interferon regulator signaling pathway in J774-Dual™ reporter cells; (**c**, **d**, and **e**): Detection of the expression levels of cytokines interferon-γ (IFN-γ), interleukin-10 (IL-10), and IFN-γ/IL-10 ratio after activation of PBMCs; (**f** and **g**): Detection of CD80/86 expression levels in PBMCs activated by r-*L. lactis*-Tα1-IFN or lipopolysaccharide (LPS, antigen-presenting cell Toll-like receptor 4 agonists). Data are presented as mean ± standard deviation. Statistical significance was set at *P < 0.05, **P < 0.01, ***P < 0.001, ****P < 0.0001

In vitro studies showed that r-*L. lactis*-Tα1-IFN lysis mixture interacted with macrophage reporter cells J774-Dual™ and significantly activated the NF-κB signaling pathway in macrophage J774-Dual™ cells compared to agonists (pam3csk4) and blank control (*P* < 0.0001; Fig. 2a). r-*L. lactis*-Tα1-IFN lysis mixture effectively stimulated the NF-κB signaling pathway in macrophage J774-Dual™ cells.

Activation of IRF pathway by r-*L. lactis*-Tα1-IFN lysis mixture was highly significant compared with wt-*L. lactis* control (*P* < 0.0001; Fig. 2b) and 2’3’-cGAMP-stimulated positive control (*P* < 0.0001; Fig. 2b). Compared to the blank control, the difference was highly significant (*P* < 0.0001; Fig. 2b). The results showed that r-*L. lactis*-Tα1-IFN lysis mixture could effectively stimulate the interferon regulatory factor (IRF) signaling pathway in macrophage J774-Dual™ cells.

### **The lysis mixture of r-*L. lactis*-Tα1-IFN activated PBMCs in vitro**

Quantitative PCR analysis of cytokines revealed that r-*L. lactis*-Tα1-IFN lysis mixture effectively stimulated the production of the cytokine IFN-γ in chicken PBMCs (Fig. 2c), and the difference between r-*L. lactis*-Tα1-IFN lysis mixture and PBS control was highly significant (*P* < 0.0001; Fig. 2c). The r-*L. lactis*-Tα1-IFN lysis mixture effectively stimulated the production of cytokine IL-10 in chicken PBMCs (Fig. 2d), and the difference between r-*L. lactis*-Tα1-IFN lysis mixture and PBS control was highly significant (*P* < 0.0001; Fig. 2d). The differences were significant, (*P* < 0.05; Fig. 2d) compared to wt-*L. lactis* control. r-*L. lactis*-Tα1-IFN effectively activated chicken PBMCs. Additionally, our study demonstrated that r-*L. lactis*-Tα1-IFN significantly upregulated the expression of CD80 and CD86 in chicken PBMCs (Fig. 2f and g). r-*L. lactis*-Tα1-IFN significantly upregulated the expression of CD80 and CD86 compared with the control group (*P* < 0.0001; Fig. 2f and g). The IFN-γ/IL-10 ratio of r-*L. lactis*-Tα1-IFN group is significantly higher than that of wt-*L. lactis* control group and blank control (*P* < 0.0001; Fig. 2e).

### **Immunopotentiation activity of the lysis mixture of r-*L. lactis*-Tα1-IFN in chickens**

**Fig. 3:**
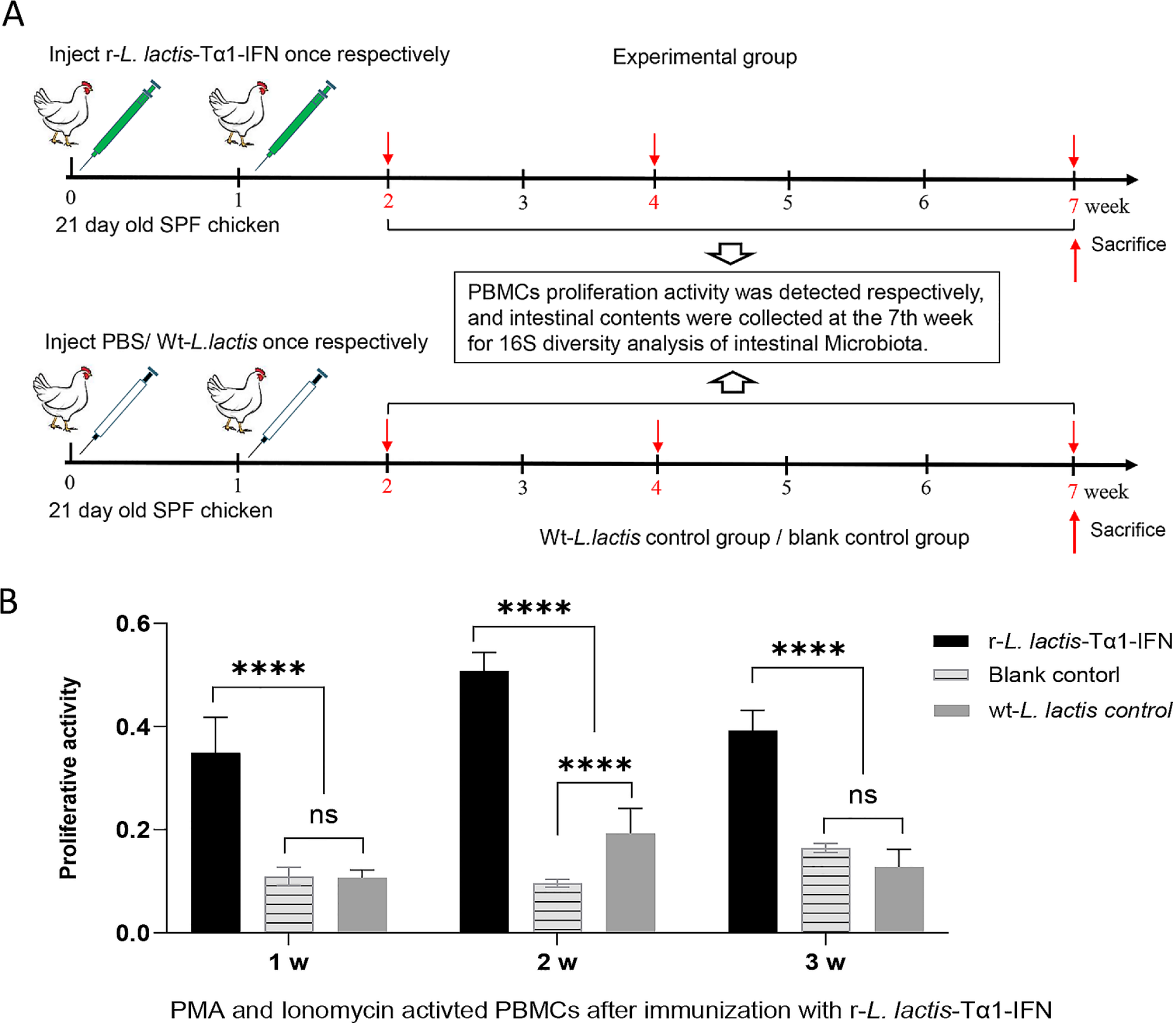
Animal experiments on recombinant r-*L. lactis*-Tα1-IFN as an immune enhancer. **(a)** Schematic diagram of animal experiments on recombinant r-*L. lactis*-Tα1-IFN as an immune enhancer; **(b)** Analysis of T lymphocyte proliferation activity of animal peripheral blood mononuclear cells after activation of PMA + Ionomycin; Data are presented as mean ± standard deviation. Statistical significance was set at *P < 0.05, **P < 0.01, ***P < 0.001, ****P < 0.0001

The results of T lymphocyte proliferative activity demonstrated the proliferative activity of PBMCs in the r-*L. lactis*-Tα1-IFN immune enhancer group was significantly higher than that of the blank control group and wt-*L. lactis* control group at the first, second and third weeks after immunization (*P* < 0.0001; Fig. 3b). Additionally, after 2 weeks of immunization, the T lymphocyte proliferative activity of PBMCs in the wt-*L. lactis* immune enhancer group was significantly higher than that of the blank control group (*P* < 0.0001; Fig. 3b), and at the first, and third weeks after immunization, the T lymphocyte proliferative activity of PBMCs in the wt-*L. lactis* immune enhancer group was not significant compared to that in the blank control group (*P* > 0.05; Fig. 3b).

**Fig. 4:**
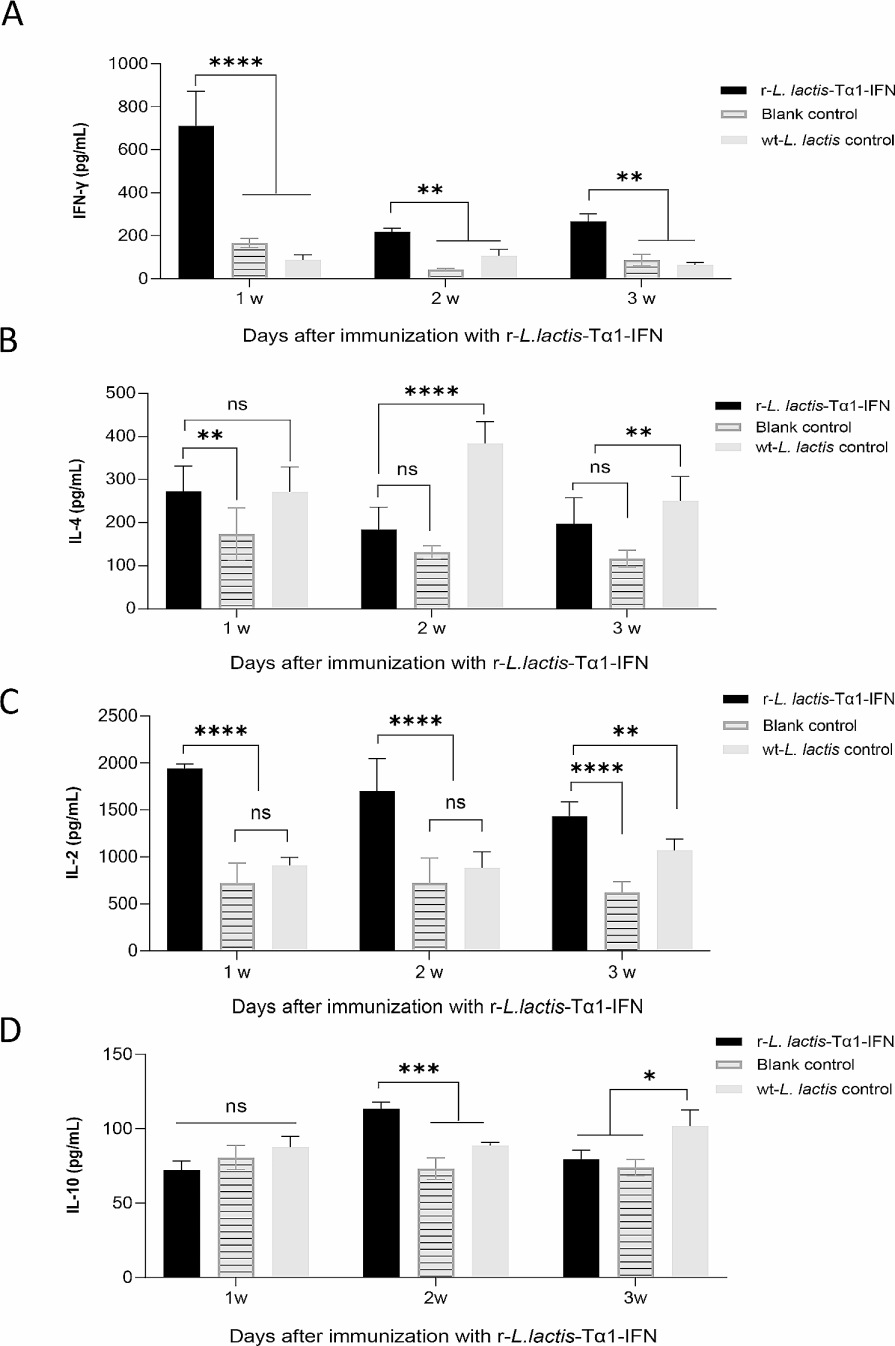
Detection of cytokines after immunization with recombinant r-*L. lactis*-Tα1-IFN. (**a**, **b**, **c** and **d**) Serum cytokines interferon-γ, interleukin (IL)-4, IL-2, and IL-10 are detected in each group at weeks 1, 2, and 3 after immunization, respectively

Cytokines detection displayed that after 1 week of immunization, the concentration of IFN-γ and IL-2 in the r-*L. lactis*-Tα1-IFN immune enhancer group was significantly higher than that in the blank control group and the wt-*L. lactis* control group (*P* < 0.0001; Fig. 4a and c). After 2 weeks of immunization, the concentration of IL-2 in the r-*L. lactis*-Tα1-IFN immune enhancer group was significantly higher than that in the control group and the wt-*L. lactis* control group (*P* < 0.0001; Fig. 4c). Furthermore, after 3 weeks of immunization, the concentration of IL-4, and IL-10 in the r-*L. lactis*-Tα1-IFN immune enhancer group was not higher than that in the blank control group and the wt-*L. lactis* control group (Fig. 4b and d).

### **The lysis mixture of r-*L. lactis*-Tα1-IFN improved the structure and composition of the intestinal microbiota in chickens**

**Fig. 5:**
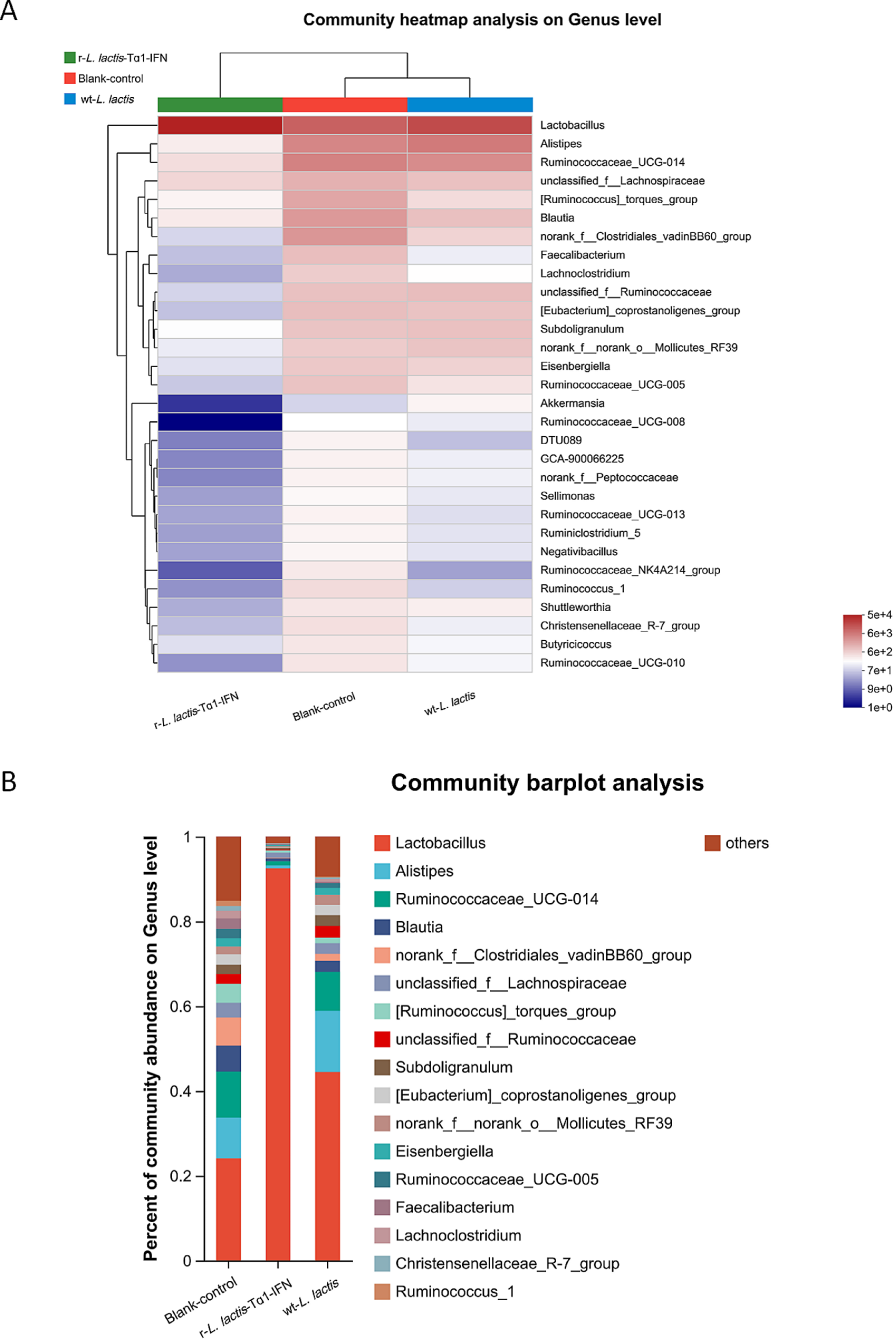
Comparative analysis of intestinal microbiota based on a heat map and bar plot. **(a)** Comparative analysis of cecal microbiota structure at the level of the first 30 genera based on heat map; **(b)** Comparative analysis of cecal microbiota structure at the level of the first 30 genera based on bar plot

**Fig. 6:**
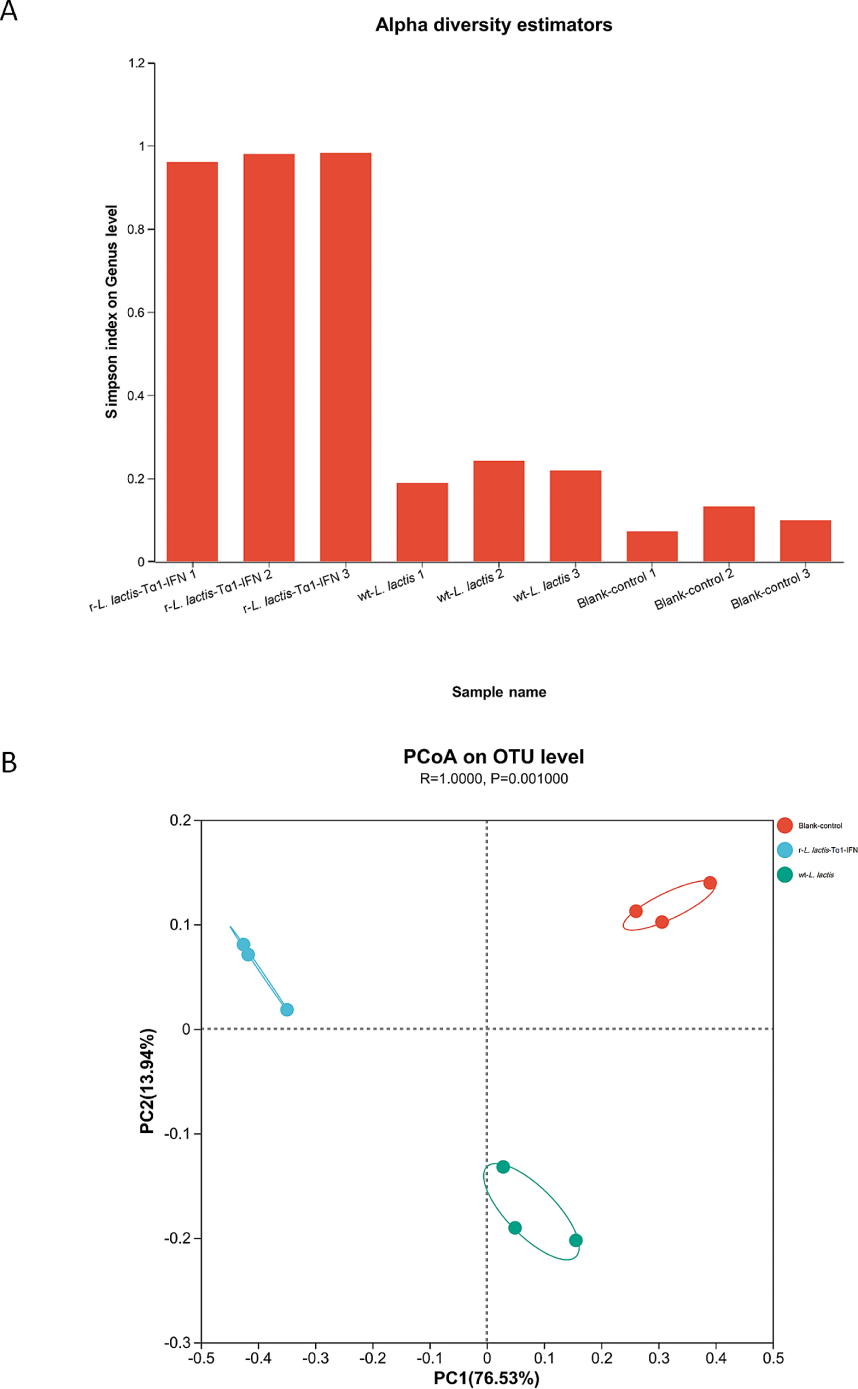
Analysis of intestinal alpha and beta microbiota diversity. **(a)** The alpha diversity index analysis of the diversity of cecal microbiota was carried out; **(b)** The similarity or difference of the composition structure of the cecal microbiota within and between groups was analyzed for Beta diversity, and the principal coordinate analysis of the cecal microbiota was performed at the OTU level

**Fig. 7:**
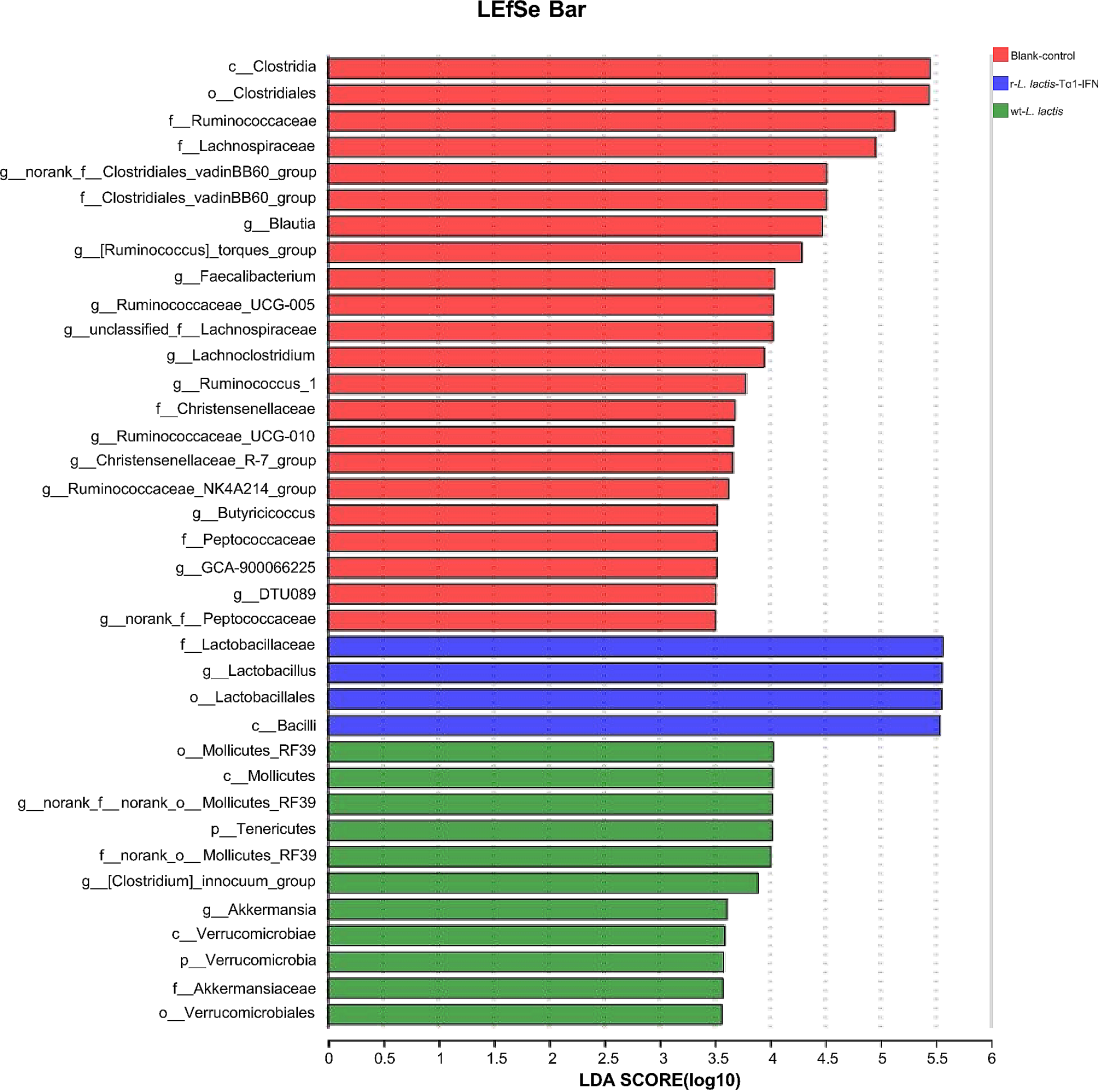
Multivariate statistical Lefse analysis of intestinal microbiota. Multivariate statistical Lefse analysis of cecal microbiota after immune recombinant r-*L. lactis*-Tα1-IFN as an immune enhancer

Heatmap analysis of the top 30 gut microbiota at the genus level demonstrated that r-*L. lactis*-Tα1-IFN significantly altered the intestinal community structure of the cecum compared with the blank control group and the wt-*L. lactis* group, and the abundance of microbiota was significantly altered in the r-*L. lactis*-Tα1-IFN group. In the r-*L. lactis*-Tα1-IFN group, beneficial probiotics, such as *Lactobacillus* were more abundant, while the abundance of *Lachnoclostridium*, *Ruminococcaceae*_UCG-008, unclassified_0_*Clostridiales*, was lower (Fig. 5a). There was a slight increase in beneficial flora in the wt-*L. lactis* group compared to the blank control group, but not as pronounced as in the r-*L. lactis*-Tα1-IFN group (Fig. 5a). Community Bar-based analysis revealed that at the genus level, sample diversity differed between r-*L. lactis*-Tα-IFN group, wt-*L. lactis* group and the blank control group, and the abundance of *Lactobacillus* increased significantly in the r-*L. lactis*-Tα1-IFN group, whereas *Ruminococcaceae*_UCG-014, *Alistipes, Blautia, norank_ f__Clostridiales*_vadinBB60_group, *[Ruminococcus]_torques*_group, unclassified_f__*Lachnospiraceae*, *Subdoligranulum*, and others decreased in abundance (Fig. 5b). When the wt-*L. lactis* group was compared with the blank control group, *Lactobacillus* increased relatively, but not as significantly as the r-*L. lactis*-Tα-IFN group (Fig. 5b). The alpha diversity index-based analysis displayed that sample diversity differed between the r-*L. lactis*-Tα1-IFN group, wt-*L. lactis* group and the blank control group, with the r-*L. lactis*-Tα1-IFN group exhibiting greater diversity than the blank control group and wt-*L. lactis* group (Fig. 6a). In addition, beta diversity analysis of the similarity or difference in the composition structure of sample communities within and between groups and principal coordinate analysis of samples at the OTU level showed that there were differences in the microbiota between the r-*L. lactis*-Tα1-IFN group, wt-*L. lactis* group and the blank group, with closer substrate distances within groups and farther substrate distances between groups, indicating significant separation of intestinal microbiota, suggesting better sampling in each group (Fig. 6b). Linear discriminant analysis (LDA) combined with effect size measurement (LEfSe) demonstrated that species with a high relative abundance in r-*L. lactis*-Tα1-IFN group was g__*Lactobacillus*, f__*Lactobacillaceae*, o__*Lactobacillales*, and c__*Bacilli*, all of which had an LDA SCORE (log10) greater than 4.5 (Fig. 7). bacteria with LDA scores > 4.0 in the wt-*L. lactis* group were mainly come from c__*Mollicutes*, o__*Mollicutes*_RF39, g__norank-f__norank_o__*Mollicutes*_RF39, p__*Tenericutes*, and f_norank_o__*Mollicutes*_RF39 (Fig. 7). while the blank control group LDA scores > 4.5 were mainly come from c_ *Clostridia*, o_ *Clostridiales*, f_ *Ruminococcaceae*, and f_*Lachnospiraceae* (Fig. 7). These results clearly show that r-*L. lactis*-Tα1-IFN increases the relative abundance of beneficial microbiota and alters the composition of the gut microbiota. In particular, the number of probiotics, such as *Lactobacillus*, significantly increased.

### **The lysis mixture of r-*L. lactis*-Tα1-IFN as an inactivated vaccine immune adjuvant**

**Fig. 8:**
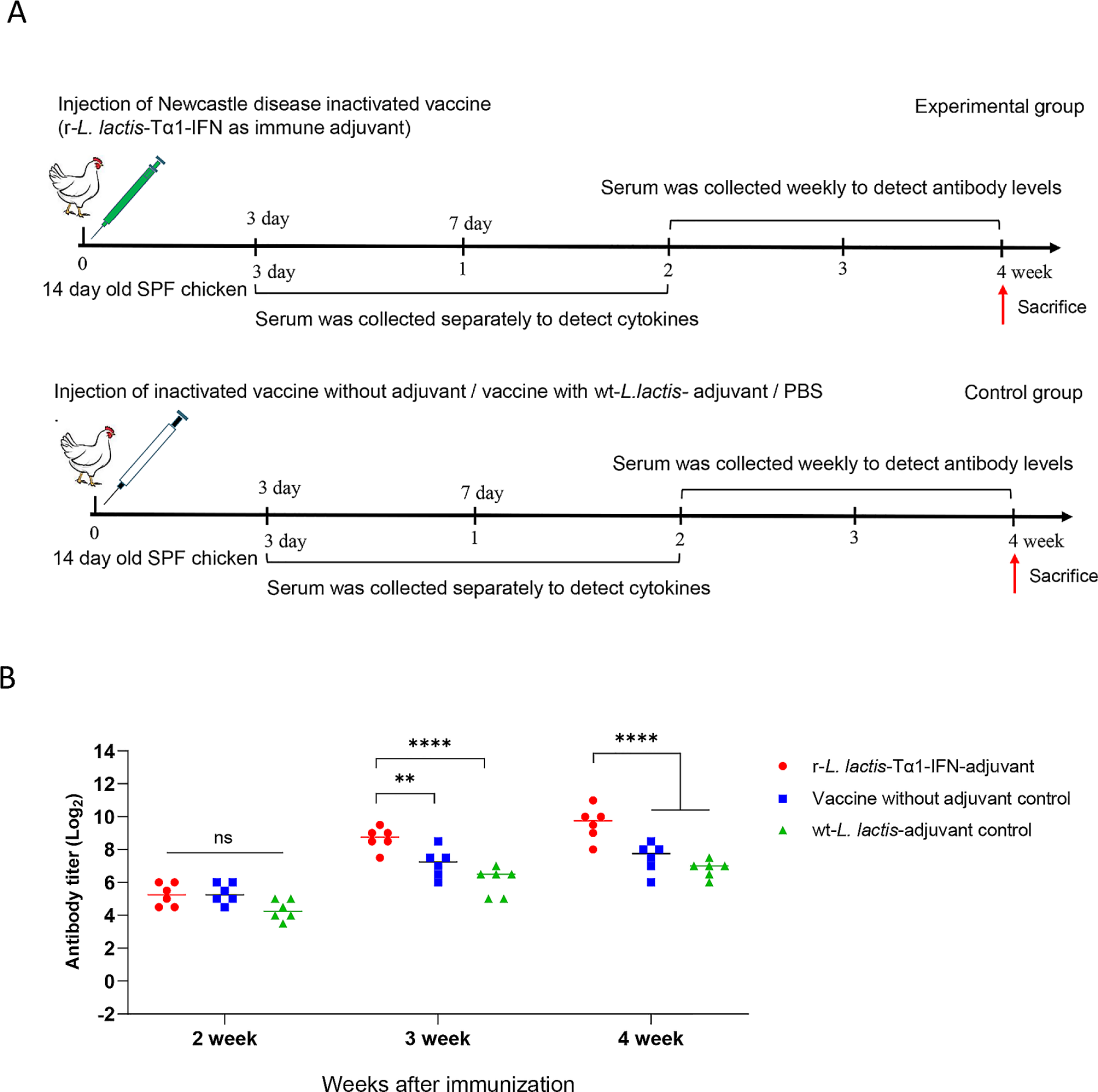
Animal experiments on recombinant r-*L. lactis*-Tα1-IFN as an immune adjuvant for Newcastle disease-inactivated vaccine. **(a)** Schematic diagram of recombinant r-*L. lactis*-Tα1-IFN as an immune adjuvant of Newcastle disease-inactivated vaccine in immunized animals; **(b)** Recombinant r-*L. lactis*-Tα1-IFN as an immune adjuvant for inactivated Newcastle disease vaccine, antibody level results in each group after 2, 3, and 4 weeks of immunization in animals. Data are presented as mean ± standard deviation. Statistical significance was set at *P < 0.05, **P < 0.01, ***P < 0.001, ****P < 0.0001

Serum antibody titers in each group demonstrated that after 2 weeks of immunization, the ND antibody titers in the r-*L. lactis*-Tα1-IFN immune adjuvant group was not significantly different from those in the vaccine control group (*P* > 0.05; Fig. 8b); however, after 3 weeks of immunization, the ND antibody titers in the r-*L. lactis*-Tα1-IFN immune adjuvant group was significantly higher than that of the vaccine control group (*P* < 0.01; Fig. 8b); and significantly higher than the wt-*L. lactis*-adjuvant control group (*P* < 0.0001; Fig. 8b), and after 4 weeks of immunization, the ND antibody titers in the r-*L. lactis*-Tα1-IFN immune adjuvant group was significantly higher than those in the vaccine control group (vaccine control) and the wt-*L. lactis*-adjuvant control group (*P* < 0.0001; Fig. 8b).

**Fig. 9:**
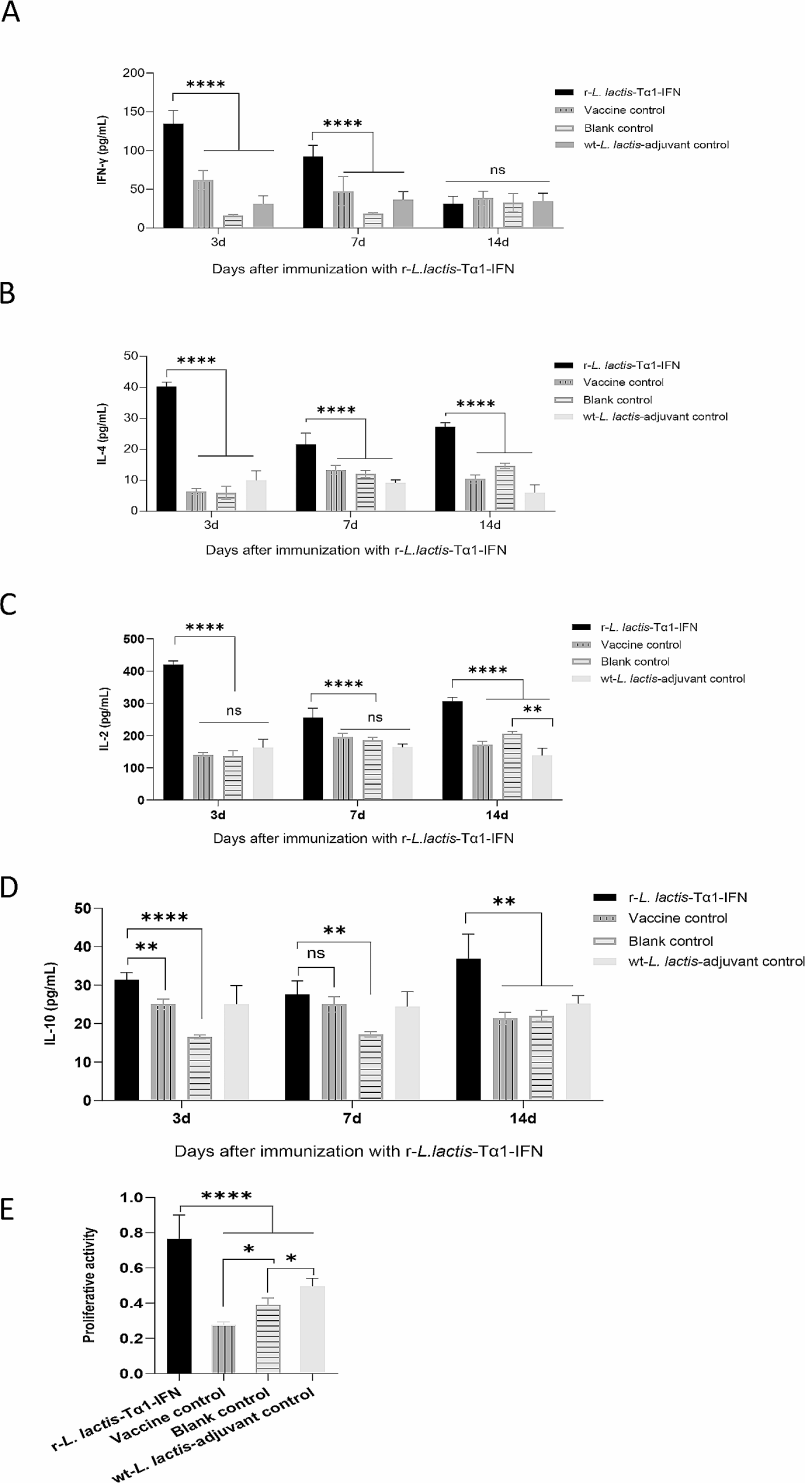
Comparison of cytokine levels and T lymphocyte proliferation activity after immunization with recombinant lactic acid bacteria r-***L. lactis***-Tα1-IFN. (**a**, **b**, **c**, and **d**) Serum cytokines interferon-γ, interleukin (IL)-4, IL-2, and IL-10 are detected in each group on post-immunization days 3, 7, and 14, respectively. (**e**) T lymphocyte proliferative activity analysis of PMA + Ionomycin activated peripheral blood mononuclear cells. Data are presented as mean ± standard deviation. Statistical significance was set at *P < 0.05, **P < 0.01, ***P < 0.001, ****P < 0.0001

ELISA results demonstrated that the concentration of IFN-γ in the r-*L. lactis*-Tα1-IFN immune adjuvant group was significantly higher than that in the vaccine control group at 3 and 7 d after the first immunization (*P* < 0.0001; Fig. 9a). and at 14 d after immunization, the concentrations of IFN-γ in the r-*L. lactis*-Tα1-IFN immune adjuvant group was no significant difference compared with the blank control group, the vaccine control group, and the wt-*L. lactis*-adjuvant control group (*P* > 0.05; Fig. 9a).

IL-2 and IL-4 in the r-*L. lactis*-Tα1-IFN immune adjuvant group were significantly higher than that in the vaccine control group, the blank control group, and the wt-*L. lactis*-adjuvant control group at 3, 7 d and 14 d after immunization (*P* < 0.0001; Fig. 9b and c),

IL-10 levels were significantly higher in the r-*L. lactis*-Tα1-IFN immunoadjuvant group than that in the vaccine control group at 3 d and 14 d after immunization (*P* < 0.01; Fig. 9d). and at 7 d after immunization, the concentrations of IL-10 in the r-*L. lactis*-Tα1-IFN immune adjuvant group was no significant difference compared with the wt-*L. lactis*-adjuvant control group (*P* > 0.05; Fig. 9d).

T lymphocyte proliferative activity assays of PBMC co-activated with Ionomycin and PMA confirmed that after 14 days of immunity, the T lymphocyte proliferative activity of r-*L. lactis*-Tα1-IFN immune adjuvant group was significantly higher than that of the vaccine control group, the wt-*L. lactis*, and blank control groups. (*P* < 0.0001; Fig. 9e).

## Discussion

In this study, the expression host strain NZ3900 was designed for food-grade applications in the nisin-controlled gene expression system (NICE®) system, which cannot be grown on lactose. However, lactose growth can be restored by providing lacF in a plasmid such as pNZ8149. The strictly NICE® system, developed at NIZO Food Research, NL, is easy to operate and has advantages for the expression of prokaryotic and eukaryotic membrane proteins. The NICE® system offers several advantages over other expression systems, including minimal endogenous and no exogenous proteases, an endotoxin-free food-grade expression system, absence of inclusion bodies and spores, tight control, and suitability for large-scale applications. In the present study, we used a lysis mixture of *Lactococcus lactis* NZ3900 as an immune adjuvant. Except for the recombinant proteins, the components of *Lactococcus lactis* did not play a statistically significant role in the immune adjuvant activity. *Lactobacillus* is believed to have a better immune adjuvant activity than *Lactococcus lactis*, and oral lysis mix of *Lactococcus lactis* or r-*L. lactis*-Tα1-IFN did not observe significant immunoadjuvant activity in our previous study (data not shown).

With the continuously expanding research in immunology and the rapid development of genetic engineering technology, researchers have applied *E. coli*, yeast, and *Streptomyces* expression systems for protein expression. Each of these expression systems has advantages and disadvantages [20–24]. *Bacillus subtilis* has biosafety properties [25] and has been rated as a biosafety (GRAS) strain by the US Food and Drug Administration. This expression system has the advantages of a mature process, no inclusion body, and easy isolation and purification of the target gene product. The disadvantage is that the promoter transcription level is low, resulting in some proteins not being expressed in large quantities, and there remain issues of unstable plasmid isolation and structural instability, cumbersome operation, low transformation efficiency, and other shortcomings that limit its application [26–29]. The yeast expression system does not produce toxins, is safe and reliable, and has good activity and stability of expressed protein products. However, the characteristics of low secretory protein ability, lack of promoters for efficient expression, and differences between post-translational processed protein products and higher eukaryotes also limit their wide application in exogenous protein production [30, 31].

In this study, a food-safe host strain (*Lactococcus lactis* NZ3900) was used as the expression host. *Lactococcus lactis* NZ3900 evolved from a strain of *Lactococcus lactis* that has been used in humans and animals for thousands of years and is currently recognized as a food-safe-grade probiotic. Nisin is a natural antimicrobial peptide that has been used for hundreds of years [32]. Therefore, the recombinant r-*L. lactis*-Tα1-IFN and the natural thymus peptide and INF completely satisfy the requirements of biosafety and green food safety.

In this study, we found that the r-*L. lactis*-Tα1-IFN induced high levels of IFN-γ, IL -2 and IL-4 production in immune cells in vivo. In the immune system, Th1 and Th2 cells belong to two different CD_4_^+^ T cell subsets that secrete two different types of cytokines and play different immunomodulatory roles. Th1 cells and Th1 cytokines mainly mediate cellular immune responses. IFN-γ is a typical Th1 cytokine, which is mainly produced in activated T cells, NK cells, activated macrophages, and a small number of NKT cells. Moreover, IFN-γ promotes cellular immune responses and the development and differentiation of lymphocytes and macrophages and inhibits the secretion of Treg cytokines. It is also a pro-inflammatory and anti-infective cytokine that plays key roles in the antiviral immune response and immunomodulation of viral and cancer immunity [33]. Th2 cells and Th2-type cytokines mainly regulate humoral immune responses [34, 35]. IL-4 is a representative cytokine secreted by Th2 cells and is mainly produced by activated T cells. IL-4 can activate T cells, B cells, and macrophages by stimulating the humoral immune response and promoting antibody production. This study demonstrated that injection of r-*L. lactis*-Tα1-IFN as an immune adjuvant in chicken produced high levels of IFN-γ, IL -2 and IL-4 after 3 d of immunization, indicating that cellular and humoral immune responses can be activated simultaneously, and injection of r-*L. lactis*-Tα1-IFN could both activate the immune response and maintain immune homeostasis.

In this study, we used r-*L. lactis*-Tα1-IFN as an immune adjuvant for the inactivated vaccine, which induced high levels of cytokine IL-2 in the organism at the first, second and third weeks after immunization. IL-2 is mainly secreted by T lymphocytes, is a key cytokine that can induce T cell proliferation, activate a variety of immune cells, and induce the proliferation and differentiation of T lymphocytes, NK cells, B cells, and monocyte macrophages [33]. These results suggest that the r-*L. lactis*-Tα1-IFN acts as an immune adjuvant and consistently promotes a high level of cellular immune response over 3 weeks.

We also performed rRNA analysis of the intestinal microbiota 16S analysis after the injection of r-*L. lactis*-Tα1-IFN. Intestinal flora 16S analysis mostly uses OTUs to analyze the composition and structure of intestinal communities, alpha and beta diversity analysis, LEfSe analysis, and other indicators for overall analysis and evaluation [36–38]. The results of this study showed that the r-*L. lactis*-Tα1-IFN injection as an immune booster or immune adjuvant could induce large changes in the composition and structure of the intestinal microbiota in the gut, and the relative reduction in the abundance of the Phylum Proteobacteria in this experiment suggested that the intestinal microbiota could maintain a relatively stable intestinal microbial community structure after injection of r-*L. lactis*-Tα1-IFN. Our results also revealed that beneficial probiotics, such as *Lactobacillus, Phylum Bacillus*, *Blautia*, and *Ruminococcus*, were more abundant, and they became the main genera of the intestinal microbiota of the cecum contents, among which the *Lactobacillus* genus played a critical role in shaping the intestinal microbiota. Some of these strains could produce antimicrobial compounds such as hydrogen peroxide and short-chain fatty acids, which inhibit pathogen invasion and help maintain intestinal health [39]. Our findings suggest that a significant increase in the abundance of *Lactobacillus* spp. improves the composition of intestinal microbiota.

Members of the order *Bacteroides* are the most abundant Gram-negative bacteria in the phylum *Bacteroides*. As the Bacteroides phylum is an important core flora in the gastrointestinal tract of mammals, a co-evolutionary relationship exists between it and the mammalian host and has a profound beneficial effect on the host immune system [40, 41]. The significant increase in the proportion of Bacteroides in our study suggests a beneficial change in the composition of the intestinal flora after r-*L. lactis*-Tα1-IFN injection.

*Blautia* is also an important core bacterial genus in the intestinal tract of animals that can inhibit pathogenic bacteria and exert anti-inflammatory effects, contributing to the alleviation of inflammatory and metabolic diseases [42]. Further, *Blautia* is also the cornerstone microbiota of the intestine that stabilizes the intestinal barrier and plays a crucial role in metabolism [43]. The results of this study showed that the injection of r-*L. lactis*-Tα1-IFN as an immune enhancer or immune adjuvant significantly increased the abundance of beneficial probiotic groups, such as *Lactobacillus, Bacteroides, Blautia*, and *Ruminococcus*; reconstructed and improved the intestinal flora of chickens; promoted the increase in probiotic abundance; and inhibited gram-negative and pathogenic bacteria. These increased beneficial microbiotas can comprehensively regulate immune response, promote immune response, and maintain immune balance and other functions through the gut-immune axis. It is of great significance for maintaining local intestinal integrity and the stability of the environment throughout the body.

In this study, natural Tα1-IFN fusion protein was expressed by food-grade *Lactococcus lactis*, and recombinant Tα1-IFN fusion protein was used as an immune enhancer or immune adjuvant. On the one hand, Tα1-IFN fusion protein demonstrated strong immunomodulatory and immune enhancement activity in vivo, which could simultaneously activate and improve cellular and humoral immune responses and maintain the body’s immune regulation homeostasis. On the other hand, injection of recombinant Tα1-IFN as an immune adjuvant or immune enhancer could significantly improve the structure and composition of the intestinal microbiota, increase the abundance of probiotics, and maintain the homeostasis of the environment in vivo. Recombinant r-*L. lactis*-Tα1-IFN has great potential for application as a vaccine immune adjuvant, and this study has important reference significance for the research and application of novel vaccine adjuvants.
